# Selling reduction versus Niggli reduction for crystallographic lattices

**DOI:** 10.1107/S2053273318015413

**Published:** 2019-01-01

**Authors:** Lawrence C. Andrews, Herbert J. Bernstein, Nicholas K. Sauter

**Affiliations:** a Ronin Institute, 9515 NE 137th Street, Kirkland, WA 98034-1820, USA; b Rochester Institute of Technology, c/o NSLS-II, Brookhaven National Laboratory, Upton, NY 11973, USA; c Lawrence Berkeley National Laboratory, 1 Cyclotron Road, Berkeley, CA 94720, USA

**Keywords:** unit-cell reduction, Delaunay, Delone, Niggli, Selling

## Abstract

The unit-cell reduction described by Selling and used by Delone (whose early publications were under the spelling Delaunay) is explained in a simple form. The transformations needed to implement the reduction are listed. The simplicity of this reduction contrasts with the complexity of Niggli reduction.

## Abstract 2

The unit-cell reduction described by Selling and used by Delone (Delaunay) is explained in a simple form.

## 1. Introduction   

The origin of crystallography was the study of minerals (Haüy, 1784 ▸). That led to the study of lattices, since it was clear that repetition underlaid the structure of crystals. In order to systematize the enumeration of lattices (unit cells), the mathematical procedures of reduction were developed to produce compact descriptions, thus providing a method to compare pairs of lattices. Fundamentally, reduction is used to place the lattice in an asymmetric unit of the space of unit cells. For some applications (*e.g.* cell clustering by pairwise comparison), the use of lattice reduction can be computationally time-consuming for large data sets. Of the known reduction methods, the Selling reduction is the least time-consuming.

Niggli (1928 ▸) and Delaunay (1933 ▸; later work used the spelling Delone) used reduction methods developed in the 19th century by Eisenstein (1851 ▸) and Selling (1874 ▸), respectively. Their original goal was to systematize the experimental determination of Bravais lattice types. Each provided tables of the characteristics of the reduced cells with their correspondence to each of the Bravais types. The first edition of *International Tables for X-ray Crystallography* originally included a section (Henry & Lonsdale, 1952 ▸) on Delone’s methods and Selling reduction and their use. Some later editions have instead included only Niggli’s method.

The initial impetus for the developments by Delone and Niggli was to determine likely Bravais lattice types based on experimental unit cells. [Probably the best display of Niggli’s methods is that by Roof (1967 ▸).] With time and the progress in crystallography, researchers realized that those tables did not always provide a simple answer due to unavoidable experimental errors in cell determination. Later, simply measuring the difference between pairs of lattices became important. Methods have been developed to cope with the resulting approximate cells [reviewed by Andrews & Bernstein (2014 ▸)].

Bravais lattice determination has been automated by several methods [see the review by Andrews & Bernstein (2014 ▸)]. However, the accumulation of databases of unit-cell parameters, often of closely similar materials, increased the need for perturbation stability. At the present time, the need is for methods that can be used to access large databases of unit-cell parameters and for cluster analysis of substantial numbers of images from serial crystallography. Bravais lattice determination is no longer the only or even the most important use of lattice-reduction methods. Now the most pressing need is for high-performance methods for lattice matching.

Andrews & Bernstein (2014 ▸, 1988 ▸) discussed Niggli reduction. In this article, we provide a complete description of the reduction of Selling that can be applied in crystallography as a time-cost-effective alternative to more complex reduction methods. Especially in procedures for handling large numbers of experimental data, reduction can be a significant portion of the processing time. Loading large databases (there are now approximately one million unit cells available) and clustering many images from serial crystallography can be a lengthy process.

One strong advantage of the use of Selling scalars is that they are homogeneous. They are all dot products and of comparable dimensions. There is another lattice representation which is also homogeneous, as seven squared lengths forming a space called **D^7^**. For completeness, we present that representation in Appendix *A* [App appa] and in the supporting information, inasmuch as literature on **D^7^** is not easily available elsewhere. The Selling representation as six scalars is computationally more efficient for database work and for clustering than the representation as seven squared lengths. Indeed, it appears to be the most efficient choice available when quantifying differences among any large number of crystallographic lattices.

## 2. The Selling scalars   

As applied to crystallography, the scalars to be reduced by Selling’s method are the dot products of the unit cell axes, in addition to the negative of their sum (a body diagonal). Labeling these **a**, **b**, **c** and **d** (**d** = −**a** − **b** − **c**), the scalars are (**b** · **c**, **a** · **c**, **a** · **b**, **a** · **d**, **b** · **d**, **c** · **d**), where *e.g.* **b** · **c** represents the dot product of the **b** and **c** axes. For the purpose of organizing these six quantities in this article, we describe them as a vector, **s**, with components *s*_1_, *s*_2_, *s*_3_, …, *s*_6_. For the purpose of Selling reduction, zero is considered to be a negative value.

## 3. The tetrahedron   

An alternative description of the scalars, due to Bravais (1850 ▸), is to consider the scalars as the labels of the edges of a tetrahedron spanned by the ends of **a**, **b**, **c** and **d** (where **d** is the negative sum of **a**, **b** and **c** as defined above). There is no preferred ordering of the four vectors, and each possible right-handed ordering generates the same lattice. Here, **a** · **b** is the label of the edge between the ends of vectors **a** and **b** *etc.* In the quote below, ‘opposite’ refers to a pair of edges of the tetrahedron across the tetrahedron from each other. This is only a formal labeling; associated with each pair of vertices, the edge between them is labeled with the dot product of the two vectors ending at those vertices.

## 4. The reduction   

Delone *et al.* (1975 ▸) state ‘Select any positive parameter of the tetrahedron and subtract it from the parameter standing on the opposite edge of the tetrahedron (the tetrahedron is at all times thought of as spatial), add it to the parameters standing on the remaining four edges, interchange the places of the obtained parameters on two of these four edges, converging to one of the ends of the original edge (it makes no difference to which), and, finally, change the sign of the positive parameter itself being considered.’

The goal of Selling reduction is to produce a set, *S*, of scalars where all elements of *S* are negative or zero. By ‘opposite’, here, is meant pairs of scalars that do not have a common element (and are on opposite edges of the Bravais tetrahedron):

**b · c** and **a · d** (*s*_1_ and *s*_4_),

**a · c** and **b · d** (*s*_2_ and *s*_5_),

**a · b** and **c · d** (*s*_3_ and *s*_6_).

For example, assuming that *s*_1_ is positive, the reduction step for *s*_1_ produces:

(−*s*_1_, *s*_2_ + *s*_1_, *s*_5_ + *s*_1_, *s*_4_ − *s*_1_, *s*_3_ + *s*_1_, *s*_6_ + *s*_1_) or

(− *s*_1_, *s*_6_ + *s*_1_, *s*_3_ + *s*_1_, *s*_4_ − *s*_1_, *s*_5_ + *s*_1_, *s*_2_ + *s*_1_).

This is continued until all six scalars are negative, known to be a ‘unique’ solution (Bravais, 1850 ▸). The reason that the choice does not matter is that the two choices are related by one of the reflections (below).

In the previous paragraph, ‘unique’ means that the list of the six scalars is unique. Their arrangement is not unique. In terms of the tetrahedron, there are 24 allowed relabelings (reflections) of the vertices. That means that, for any reduced cell, there are 24 reflections [permutations of the scalars correspond to permutations of (**a**, **b**, **c**, **d**)] that are all ‘reduced’.

Finally, as a check on the process and on the correctness of the lattice, the negative sum of the six scalars must decrease in each reduction step.

If we define the six-dimensional space of scalars as **S^6^** and the full set of Selling reduction operations as matrices on **S^6^**, then two alternative matrices per scalar being reduced are given by:

For **b** · **c** = 0 boundary: 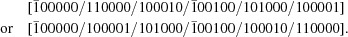



For **a** · **c** = 0 boundary: 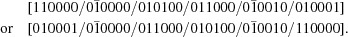



For **a** · **b** = 0 boundary: 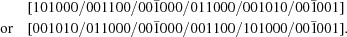



For **a** · **d** = 0 boundary: 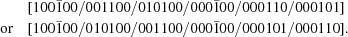



For **b** · **d** = 0 boundary: 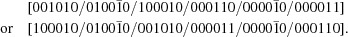



For **c** · **d** = 0 boundary: 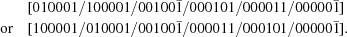
Note that the second of each pair is just a permutation of the first, so we only need the first of each pair for reduction.

We include below a pseudocode implementation of the reduction. Here, ReduceTheLargestScalar applies the corresponding operation from the list of matrices above.[Chem scheme1] 
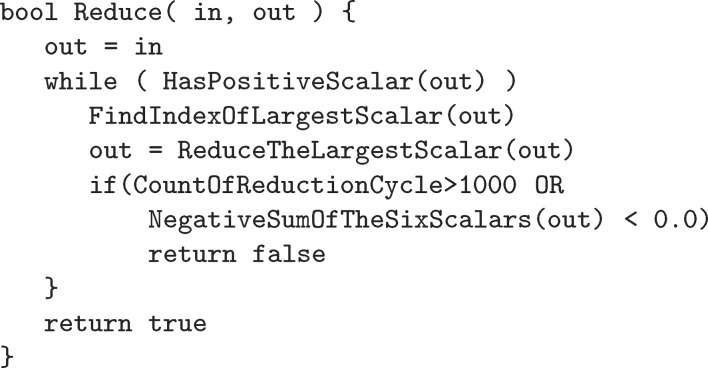


**Figure 1:**
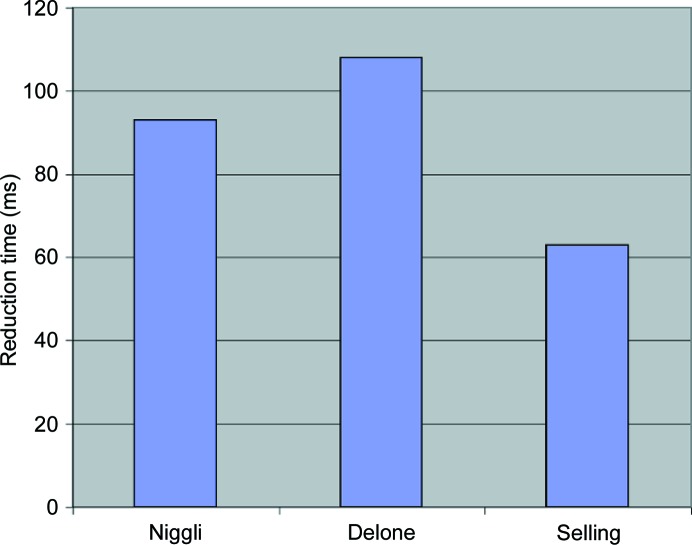
Reduction timing for 89539 unit cells taken from the Protein Data Bank (milliseconds). The times given are for primitive cells.

Experience has shown that Selling reduction is faster to execute than Niggli reduction (see Fig. 1 ▸). In many applications it is important to reduce all cells before processing. A large fraction of the cells to be considered have already been reduced before the application is run, but reduction is so important that the reduction methods are applied to all cells to at least verify that they have been reduced. Even for cells that have already been reduced, the difference in timing between ‘Niggli’ and ‘Selling’ is due to the difference in complexity for checking whether reduction is complete. The simple pseudocode above can be contrasted with the more complex algorithm for Niggli reduction [see Gruber (1973 ▸) and Andrews & Bernstein (1988 ▸)]. In addition, when working in Selling space, this same difference in complexity of reduction is reflected in a difference in the number of boundary polytopes for the fundamental region, which means that applications such as clustering and cell database searching will be faster when working with Selling-reduced cells. For example, when the cell database program *SAUC* (McGill *et al.*, 2014 ▸) is modified to use Selling reduction, a search of half a million PDB (Protein Data Bank) and CSD (Cambridge Structural Database) cells for the nearest 500 cells to 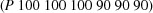 runs in 137 s CPU time and 8 s real time on a 12-core AMD Threadripper for Niggli reduction, but the same search runs in 52 s CPU time and 4 s real time for Selling reduction.

## 5. Difficulties in applying Selling reduction to the methods of Delaunay (1933 ▸)   

Of the available cell-reduction methods, Selling reduction has the fastest performance. As X-ray detectors become faster and data collection moves to higher and higher speeds, the performance of data-analysis pipelines also needs to be improved. In any system, the total system performance will not improve until the last bottleneck is removed, and in serial crystallography there are many bottlenecks to be addressed. The choice of reduction method is an important parameter to consider in this regard. As we have shown, Selling reduction as considered by Delone has much to recommend it, yet the coders of many current applications, especially for Bravais lattice identification, have favored Niggli reduction over Delone’s methods because of the issues to be discussed in the paragraphs below. Clearly, Delone’s methods are not completely forgotten, as Oishi-Tomiyasu (2012 ▸) used both Niggli and Delone methods. The solution of these problems is best dealt with algebraically by considering a lattice to be represented by a point in a vector space. This topic will be addressed in a forthcoming article.

First, the identification of lattice types is usually described in terms of matching a reduced set of scalars to one of the pictures of the 24 different Bravais tetrahedra (Delaunay, 1933 ▸) corresponding to the various lattice types (such as body-centered cubic *etc.*). This is a complex step: the user must relabel the axes of his/her own lattice picture to agree with each of the types (equivalent to choosing one or more of the 24 reflections of his/her picture to match the orientation of those of the 24 types that seem possible).

Second, the user must make decisions about how close to zero each scalar is. Each zero or near zero generates additional decisions that must be made. Further, the user may need to make a choice as to whether a near-zero value (negative after reduction) is so close to zero that another reduction should be done with that value considered positive.

Third, several of the reflections might give similar matches to a picture, and there may always be multiple matches (for instance, all cubic cells will match some orthorhombic cells).

None of these issues can outweigh the performance gains of Selling reduction over Niggli reduction in clustering and cell-database use, but, as noted above, they need to be addressed for other applications such as lattice identification, so we will not have to deal with two very different views of the same lattice in pipelines of applications. Especially when visualization rather than just computation is involved in lattice identification, the conversion from Niggli reduction back to Selling reduction to gain performance can be a complex undertaking. This will have to be addressed one application at a time in the future.

## 6. Related literature   

For additional literature relating to the supporting information, see Minkowski (1905 ▸) and Buerger (1960 ▸).

## Supplementary Material

Appendix B. DOI: 10.1107/S2053273318015413/ae5054sup1.pdf

## Appendix A A seven-space representation of lattices based on sorted Delone reduction

We review the relationship between Niggli reduction and Delone reduction and describe a representation due to Delaunay (later known as Delone) of cells in a seven-dimensional space within which the fundamental unit including sorting is convex and equivalent to the conventional representations.

### A1. Notation

{...} an unordered ensemble

[...] an ordered list

||**x**|| the norm of vector **x**

**a**, **b**, **c** three cell-edge vectors giving a unit cell of a lattice

**d** = −**a** − **b** − **c** the negative of the main cell body diagonal, the fourth vector for Delone reduction

[*g*_1_, *g*_2_, *g*_3_, *g*_4_, *g*_5_, *g*_6_] = [||**a**||^2^, ||**b**||^2^, ||**c**||^2^, 2**b** · **c**, 2**a** · **c**, 2**a** · **b**] the **G** ^**6**^ vector of [**a**, **b**, **c**]

[*P*, *Q*, *R*, *S*, *T*, *U*] = [*s*_23_, *s*_13_, *s*_12_, *s*_14_, *s*_24_, *s*_34_] = [**b** · **c**, **a** · **c**, **a** · **b**, **a** · **d**, **b** · **d**, **c** · **d**] the Selling (Delaunay, Delone) scalars of [**a**, **b**, **c**, **d**]

[*d*_1_, *d*_2_, *d*_3_, *d*_4_, *d*_5_, *d*_6_, *d*_7_] = [||**a**||^2^, ||**b**||^2^, ||**c**||^2^, ||**d**||^2^, ||**b** + **c**||^2^, ||**a** + **c**||^2^, ||**a** + **b**||^2^] = [||**a**||^2^, ||**b**||^2^, ||**c**||^2^, ||**d**||^2^, ||**d** + **a**||^2^, ||**d** + **b**||^2^, ||**d** + **c**||^2^] the **D** ^**7**^ vector of [**a**, **b**, **c**, **d**]

### A2. Delone reduction

Given three cell-edge vectors **a**, **b**, **c**, that cell is called Delone-reduced if the four vectors (the Bravais tetrahedron; Delaunay, 1933 ▸) **a**, **b**, **c**, **d** = −**a** − **b** − **c**, numbered 1 to 4, all form right angles or obtuse angles relative to one another. In terms of **G** ^**6**^ (Andrews & Bernstein, 1988 ▸), given a cell described by [*g*_1_, *g*_2_, *g*_3_, *g*_4_, *g*_5_, *g*_6_], converting from the Selling (1874 ▸) scalar **S** ^**6**^ [*P*, *Q*, *R*, *S*, *T*, *U*] notation for the inner products of Henry & Lonsdale (1952 ▸), based on Ito (1950 ▸) and Delaunay (1933 ▸), the six doubled inner products among the Bravais tetrahedron vectors are:

equivalently

For a Delone-reduced cell, all the expressions (*A*.1) through (*A*.6) must be zero or negative. This is equivalent to saying that all the cell angles must be obtuse or right angles. No acute angles are permitted.

Patterson & Love (1957 ▸) used a slightly different notation and presented a very efficient algorithm for Delone reduction.

To a minimal extent, in the past one level of ambiguity in presentation was removed by imposing various symmetry-dependent orderings. We go further in this appendix by imposing a strict ordering on the lengths of the tetrahedron vectors ||**a**|| ≤ ||**b**|| ≤ ||**c**|| ≤ ||**d**||. This ordering, while not essential to doing the reduction, clarifies the presentation of each cell.

### A3. Converting from the **G^6^** or **E^3^** representation of a Niggli-reduced cell to Delone-reduced

It is possible to achieve Delone reduction by first doing Niggli reduction. See Appendix *B* Section B.1 in the supporting information for a summary of the Niggli conditions.

Allmann (1968 ▸) presented the transformation from a Buerger-reduced cell to a Delone-reduced cell. We restate that algorithm in full detail and then specialize it to deal with the Niggli-reduction conditions.

Most of the Niggli conditions are not relevant to conversion from Niggli reduction to Delone reduction. The relevant Niggli conditions can be stated as:

and *g*_{4,5,6}_ are all strictly positive or all less than or equal to zero.

#### A3.1. The Niggli-reduced − − − case

If we have *g*_{4,5,6}_ all less than or equal to zero, examine each element of expressions (*A*.1) through (*A*.6):

the Niggli reduction case of *g*_{4,5,6}_ all less than or equal to zero is already Delone-reduced. Recall that, for these  Niggli-reduced cells,

#### A3.2. The Niggli-reduced + + + cases

Now consider the remaining case of *g*_{4,5,6}_ all greater than zero. For these + + + Niggli-reduced cells,

At least one of *g*_{4,5,6}_ is minimal. It is possible for more than one to be minimal.

#### A3.3. The Niggli-reduced + + +, *g*_6_ minimal case

Suppose *g*_6_ ≤ *g*_4_, *g*_6_ ≤ *g*_5_. In this case consider the Bravais tetrahedron

The first two components are of lengths ||**a**|| and ||**b**|| from the Niggli cell. Both lengths ||− **c** + **b**|| and ||**c** − **a**|| are greater than or equal to ||**c**||. If either were smaller, **a**, **b**, **c** would not be Niggli-reduced. There are two **G** ^**6**^ vectors to consider, using the shorter of the last two vectors in place of **c**; the remaining vector becomes the fourth tetrahedron edge:

which are  vectors in this case, and the elements of expressions (*A*.1) through (*A*.6) are

respectively, all of which are less than or equal to zero.

#### A3.4. The Niggli-reduced + + +, *g*_5_ minimal case

Suppose *g*_5_ ≤ *g*_4_, *g*_5_ ≤ *g*_6_. In this case consider the Bravais tetrahedron

The first and third components are of lengths ||**a**|| and ||**c**||, respectively, from the Niggli cell.  =  ≥  and  =  ≥ . It is possible that  ≥ , in which case **c** will replace **b** and the smaller of **b** − **a** and **c** − **b** will replace **c**. Otherwise just the smaller of **b** − **a** and **c** − **b** will replace **c**. In any of these cases, there are two **G** ^**6**^ vectors to consider, using either the second or fourth component in addition to **a** and **c**,

which are  vectors in this case, and the elements of expressions (*A*.1) through (*A*.6) are

respectively, all of which are less than or equal to zero.

#### A3.5. The Niggli-reduced + + + , *g*_4_ minimal case

Suppose *g*_4_ ≤ *g*_5_, *g*_4_ ≤ *g*_6_. In this case consider the Bravais tetrahedron

The second and third components are of lengths ||**b**|| and ||**c**||, respectively, from the Niggli cell. The length of **b** − **a** is greater than or equal to the length of **b**. The length of **a** − **c** is greater than or equal to the length of **c**. Therefore the three shortest edges of the Bravais tetrahedron will be some combination of −**b**, ||**c**|| and the shorter of **b** − **a** and **a** − **c**, so there are two **G** ^**6**^ vectors to consider,

which are  vectors in this case, and the elements of expressions (*A*.1) through (*A*.6) are

respectively, all of which are less than or equal to zero.

Note that in all cases for a Bravais tetrahedron, the three shortest edge vectors are either from a  Niggli cell, or, in the case of a Bravais tetrahedron derived from a  Niggli cell, two of the three shortest edges are from that Niggli cell with the direction of one edge reversed, and the third of the shortest edges is a face diagonal from a face involving the third Niggli cell edge.

### A4. The effects of perturbations

Perturbations of a cell can cause exchanges of edges with face diagonals or body diagonals. Because a Delone cell only has obtuse (or right) angles, the diagonals produced by sums are closer in length to the original edges than those involving differences. Therefore, in many cases, Delone reduction of a cell close to

will use the additive face and body diagonals

### A5. The seven-dimensional Delone space **D^7^**

Consider the Bravais tetrahedron **a**, **b**, **c**, **d** = −**a** − **b** − **c**.

If we consider only lengths, then the total ensemble of seven unique lengths resulting from the Bravais tetrahedron and the additive face and body diagonals is

Taking squares of these lengths gives a seven-vector defined by Delone *et al.* (1975 ▸) in a space we call **D** ^7^ for Delone seven-space.

In Appendix *B* (supporting information), the necessary and sufficient conditions for a well defined cell to be Delone-reduced are given and the fundamental region of points in seven-space satisfying those conditions is shown to be convex with seven five-dimensional boundaries.
