# Supplementary material for: Selling reduction versus Niggli reduction for crystallographic lattices
Source: Acta Crystallogr A Found Adv. 2019 Jan 1;75(Pt 1):115–20. doi: 10.1107/S2053273318015413 (PMC6302928; doi:10.1107/S2053273318015413)
Supplement: Supplementary file 1 [file a-75-00115-sup1.pdf]

Volume 75 (2019)

Supporting information for article:

Selling reduction versus Niggli reduction for crystallographic lattices

Lawrence C. Andrews, Herbert J. Bernstein and Nicholas K. Sauter

Appendix B
A Seven-Space Representation of Lattices Based on Sorted Delone Reduction – Supplementary Material

For notation and background, see the main paper sections A.1 and 1.

We review the relationship between Niggli reduction and Delone reduction and present a representation of Delone-reduced cells in a seven-dimensional space of squared lengths, $\mathbf{D^7}$, as an alternative to the six-dimensional $\mathbf{S^6}$ space Selling inner products, within which the fundamental unit including sorting is convex and equivalent to the conventional representations.

See A.2 for Delone (Delaunay) reduction. Compare this to the more complex Niggli conditions B.1. Next we turn to the structure of $\mathbf{D^7}$.

B.1. The Niggli Conditions

The Niggli-reduced cell of a lattice is a unique choice from among the infinite number of alternate cells that generate the same lattice (Niggli, 1928). A Buerger-reduced cell, which is equivalent to a Minkowski-reduced cell (Minkowski, 1905) for a given lattice is any cell that generates that lattice, chosen such that no other cell has shorter cell edges (Buerger, 1960). Even after allowing for the equivalence of cells in which the directions of axes are reversed or axes of the same length are exchanged, there can be up to five alternate Buerger-reduced cells for the same lattice (Gruber, 1973). The Niggli conditions allow the selection of a unique reduced cell for a given lattice from among the alternate Buerger-reduced cells for that lattice.

Niggli reduction consists of converting the original cell to a primitive one and then alternately applying two operations: conversion to standard presentation and reduc-

tion (Andrews & Bernstein, 1988). The convention for meeting the combined Buerger and Niggli conditions is based on increasingly restrictive layers of constraints:

If $g_1 < g_2 < g_3$, $|g_4| < g_2$, $|g_5| < g_1$, $|g_6| < g_1$ and either $g_{\{4,5,6\}} > 0$ or $g_{\{4,5,6\}} \leq 0$ then we have a Niggli-reduced cell, and we are done.

The remaining conditions are imposed when any of the above inequalities becomes an equality or the elements of $g_{\{4,5,6\}}$ are not consistently all strictly positive or are not consistently all less than or equal to zero.

The full set of combined Niggli conditions, in addition to those for the cell edge lengths being minimal, is:

require $0 \leq g_1 \leq g_2 \leq g_3$

if $g_1 = g_2,$ then require $|g_4| \leq |g_5|$

if $g_2 = g_3,$ then require $|g_5| \leq |g_6|$

require $\{g_4 > 0$ and $g5 > 0$ and $g6 > 0\}$

or require $\{g_4 \leq 0$ and $g_5 \leq 0$ and $g6 \leq 0\}$

require $|g_4| \leq g_2$

require $|g_5| \leq g_1$

require $|g_6| \leq g_1$

require $g_3 \leq g_1 + g_2 + g_3 + g_4 + g_5 + g_6$

if $g_4 = g_2,$ then require $g_6 \leq 2g_5$

if $g_5 = g_1,$ then require $g_6 \leq 2g_4$

if $g_6 = g_1,$ then require $g_5 \leq 2g_4$

if $g_4 = -g_2,$ then require $g_6 = 0$

if $g_5 = -g_1,$ then require $g_6 = 0$

if $g_6 = -g_1$, then require $g_5 = 0$

if $g_3 = g_1 + g_2 + g_3 + g_4 + g_5 + g_6$, then require $2g_1 + 2g_5 + g_6 \leq 0$

The $\mathbf{G^6}$ transformations associated with each of these steps are enumerated in (Andrews & Bernstein, 1988). Application of these transformations must be repeated until all conditions are satisfied.

B.2. The 7-Dimensional Delone Space $\mathbf{D^7}$

Consider the Bravais tetrahedron $\mathbf{a}$, $\mathbf{b}$, $\mathbf{c}$, $\mathbf{d} = -\mathbf{a} - \mathbf{b} - \mathbf{c}$

If we consider only lengths, then the total ensemble of seven unique lengths resulting from the Bravais tetrahedron and the additive face and body diagonals is

$$\{||\,\mathbf{a}\,||,\ \ ||\,\mathbf{b}\,||,\ \ ||\,\mathbf{c}\,||,\ \ ||\,\mathbf{d}\,||,\ \ ||\,\mathbf{b}+\mathbf{c}\,||,\ \ ||\,\mathbf{a}+\mathbf{c}\,||,\ \ ||\,\mathbf{a}+\mathbf{b}\,||\,\}$$

Taking squares of these lengths gives a seven-vector defined by (Delone *et al.*, 1975) in a space we call $\mathbf{D^7}$ for Delone 7-space:

$$[d_1 = ||\,\mathbf{a}\,||^2,\ \ d_2 = ||\,\mathbf{b}\,||^2,\ \ d_3 = ||\,\mathbf{c}\,||^2,\ \ d_4 = ||\,\mathbf{d}\,\,||^2,$$

$$d_5 = ||\,\mathbf{b}+\mathbf{c}\,||^2,\ \ d_6 = ||\,\mathbf{a}+\mathbf{c}\,||^2,\ \ d_7 = ||\,\mathbf{a}+\mathbf{b}\,||^2]$$

$$= [d_1 = ||\,\mathbf{a}\,||^2,\ \ d_2 = ||\,\mathbf{b}\,||^2,\ \ d_3 = ||\,\mathbf{c}\,||^2,\ \ d_4 = ||\,\mathbf{d}\,||^2,$$

$$d_5 = ||\,\mathbf{a}+\mathbf{d}\,||^2,\ \ d_6 = ||\,\mathbf{b}+\mathbf{d}\,||^2,\ \ d_7 = ||\,\mathbf{c}+\mathbf{d}\,||^2]$$

$$= [g_1,\ \ g_2,\ \ g_3,\ \ g_1 + g_2 + g_3 + g_4 + g_5 + g_6,$$

$$g_2 + g_3 + g_4,\ \ g_1 + g_3 + g_5,\ \ g_1 + g_2 + g_6]$$

$$= [-Q - R - S,\ \ -P - R - T,\ \ -P - Q - U,\ \ -S - T - U, -Q - R - T - U,$$

$$-P - R - S - U,\ \ -P - Q - S - T]$$

and

$$P = -d_2/2 - d_3/2 + d_5/2$$

$Q = d_2/2 + d_4/2 - d_5/2 - d_7/2$

$R = -d_1/2 - d_2/2 + d_7/2$

$S = -d_1/2 - d_4/2 + d_5/2$

$T = d_1/2 + d_3/2 - d_5/2 - d_7/2$

$U = -d_3/2 - d_4/2 + d_7/2$

In $\mathbf{D^7}$ the Delone reduced cells are defined by:

$$d_1,\ \ d_2,\ \ d_3,\ \ d_4,\ \ d_5,\ \ d_6,\ \ d_7 > 0 \tag{B.1}$$

$$d_1 + d_2 + d_3 + d_4 - d_5 - d_6 - d_7 = 0 \tag{B.2}$$

$$d_1 \leq d_2 \leq d_3 \leq d_4 \tag{B.3}$$

$$d_5 \leq d_2 + d_3 \tag{B.4}$$

$$d_5 \leq d_1 + d_4 \tag{B.5}$$

$$d_6 \leq d_1 + d_3 \tag{B.6}$$

$$d_6 \leq d_2 + d_4 \tag{B.7}$$

$$d_7 \leq d_1 + d_2 \tag{B.8}$$

$$d_7 \leq d_3 + d_4 \tag{B.9}$$

$$d_5 \geq d_2 - d_3 \tag{B.10}$$

$$d_5 \geq d_1 - d_4 \tag{B.11}$$

$$d_6 \geq d_1 - d_3 \tag{B.12}$$

$$d_6 \geq d_2 - d_4 \tag{B.13}$$

$$d_7 \geq d_1 - d_2 \tag{B.14}$$

$$d_7 \geq d_3 - d_4 \tag{B.15}$$

Boundaries may be defined by equalities in the above relationships.

As we will show, the conditions B.1 through B.15 are necessary and sufficient for a well-defined cell to be Delone reduced, and for the fundamental region of points in seven-space satisfying those conditions to be convex, with seven 5-dimensional boundaries.

B.3. Comments on projectors and boundaries

All the currently accepted reduction processes depend on obeying constraining linear inequalities. Such inequalities determine boundaries of the space of reduced cells. Because they are linear, the results are linear polytopes defining boundaries of the fundamental region of reduced cells. The highest dimension polytopes completely determine the shape of the fundamental region. For example. the highest order boundaries in $\mathbf{G^6}$ (which is a 6-dimensional space) are 5-dimensional polytopes. The lower dimensional boundary polytopes are all the result of the intersections of the higher dimensional polytopes.

Given a unit cell, the distance from that cell to another cell depends on whether we draw a line directly between the two cells or look at lines that are interrupted by boundaries. Metrics in spaces of reduced cells depend on an understanding of where each cell stands in relation to each boundary. For that we need projection matrices that project from a cell to the nearest cell in a boundary. The boundaries can be determined entirely algebraically; however it is more efficient to start with computational experiments that probe all boundaries and reveal which ones are more

populated and likely to be higher dimensional and therefore more fundamental to this necessary understanding.

The boundaries presented here were discovered first by Monte Carlo experiments and then by confirming algebraic analysis using the reduction inequalities that fundamentally determine the boundaries.

The process of finding projectors began by randomly generating a large number of vectors satisfying the boundary conditions. For one of the boundaries, a group of vectors that is sufficient to span the boundary polytope is generated. Treating the vectors as an m by n matrix (n vectors of m dimension), singular value decomposition gives one the eigenvectors that span the boundary polytope and those eigenvectors serve as the projector. A simpler confirming method is to take powers of the product of the m by n matrix and its transpose.

For distance calculations, the "perp", the unit matrix minus the projector, is also needed. This is represented in the equations by the $^{\perp}$ symbol.

B.4. Necessity of conditions B.1 through B.15

The necessity of B.1 follows from the definition of each of d_1 through d_7 as a length, which, as norms, must be non-negative. The zero cases can only arise from a tetrahedron with a zero edge in the first four cases, or a 180 degree angle in the last three cases.

We show the necessity of B.2 from the representations in terms of $\mathbf{G^6}$ components.

$$d_1 + d_2 + d_3 + d_4 - d_5 - d_6 - d_7$$

$$= g_1 + g_2 + g_3 + g_1 + g_2 + g_3 + g_4 + g_5 + g_6$$

$$-g_2 - g_3 - g_4 - g_1 - g_3 - g_5 - g_1 - g_2 - g_6$$

$$= (1 + 1 - 1 - 1) * g_1 + (1 + 1 - 1 - 1) * g_2 + (1 + 1 - 1 - 1) * g_3$$

$$+(1-1)*g_4+(1-1)*g_5+(1-1)*g_6=0$$

Conditions B.4 through B.9 are just a restatement of the Delone reduction condition of obtuse or right angles among the four cell edges, and thus are necessary.

Finally, the remaining conditions follow from the Cauchy-Schwarz inequality and from the tetrahedron cell edge length ordering we have chosen. For example

$$|\mathbf{b}\cdot\mathbf{c}| \leq ||\mathbf{b}||||\mathbf{c}|| \leq ||\mathbf{c}||^2$$

$$\implies -g_4 \leq 2g_3 \implies -g_3 \leq g_3+g_4 \implies g_2-g_3 \leq g_2+g_3+g_4$$

which is equivalent to B.10

The necessity of all B.10 through B.15 follows similarly.

B.5. Sufficiency of conditions B.1 through B.15

In order to show that conditions B.1 through B.15 are sufficient to define Delone reduction, we need to map from $\mathbf{D^7}$ to $\mathbf{G^6}$ and verify that the left-hand sides in A.1 through A.6 are all less than or equal to zero when B.1 through B.15 are satisfied.

Given a cell $d=(d_1,d_2,d_3,d_4,d_5,d_6,d_7)\in\mathbf{D^7}$, we define the mapping from $\mathbf{D^7}$ to $\mathbf{G^6}$ by:

$$\mathbf{D^7\_to\_G^6}(d_1,d_2,d_3,d_4,d_5,d_6,d_7)$$

$$=(d_1,d_2,d_3,d_5-d_2-d_3,d_6-d_1-d_3,d_7-d_1-d_2)$$

The requirement that A.1 through A.3 be less than or equal to zero is satisfied by applying B.4, B.6, and B.8. The requirement that A.4 through A.6 be less than or equal to zero can be seen to be satisfied by combining condition B.2 with one of conditions B.5, B.7 or B.9. For example, A.4 is equivalent to

$$0 \geq -2d_1-(d_7-d_1-d_2)-(d_6-d_1-d_3)$$

$$=-d_7+d_2-d_6+d_3$$

applying B.2 by subtracting it

$$= -d_7 + d_2 - d_6 + d_3 - (d_1 + d_2 + d_3 + d_4 - d_5 - d_6 - d_7)$$

$$= -d_1 - d_4 + d_5$$

which is equivalent to condition B.5. The other conditions are similarly satisfied. Note that we have not used all of B.1 through B.15 The system has strong redundancies. This is inherent because we are using a seven-dimensional representation of a six-dimensional space. We do this because it changes complex mappings involving the body diagonals into simple permutations.

B.6. The Seven 5-D Boundaries Polytopes of $\mathbf{D^7}$

The full 7-dimensional space is projected to a 6-dimensional space by the linear constraint B.2. We start the exploration of this space by identifying the 5-dimensional boundary polytopes that result from considering one equality in the above relationships at a time.

B.7. Cases 1, 2 and 3: Equal Bravais tetrahedron Edge Lengths

These cases arise when two Bravais tetrahedron edges have equal lengths, the equality cases in expression B.3. Consider for example $d_1 = d_2$. The boundary transformation would be based on exchanging $\mathbf{a}$ and $\mathbf{b}$, but that simple exchange would reverse the handedness of the cell, so we also negate all resulting edges to restore the handedness.

B.7.1. Case 1 $d_1 = d_2$, $Q + S = P + T$, $||\,\mathbf{a}\,||^2 = ||\,\mathbf{b}\,||^2$, $\mathbf{a} \rightarrow -\mathbf{b}$, $\mathbf{b} \rightarrow -\mathbf{a}$, $\mathbf{c} \rightarrow -\mathbf{c}$, $\mathbf{d} \rightarrow \mathbf{a} + \mathbf{b} + \mathbf{b} = -\mathbf{d}$

$$MD_1 =$$

(**01**00000/**10**00000/0010000/0001000/0000**01**0/0000**10**0/0000001)

$PD_1 =$

$$\left(\frac{5}{14}\frac{5}{14}\frac{\bar{1}}{7}\frac{\bar{1}}{7}\frac{1}{7}\frac{1}{7}\frac{1}{7}/\frac{5}{14}\frac{5}{14}\frac{\bar{1}}{7}\frac{\bar{1}}{7}\frac{1}{7}\frac{1}{7}\frac{1}{7}/\frac{\bar{1}}{7}\frac{\bar{1}}{7}\frac{6}{7}\frac{\bar{1}}{7}\frac{1}{7}\frac{1}{7}\frac{1}{7}/\frac{\bar{1}}{7}\frac{\bar{1}}{7}\frac{\bar{1}}{7}\frac{6}{7}\frac{1}{7}\frac{1}{7}\frac{1}{7}/\frac{1}{7}\frac{1}{7}\frac{1}{7}\frac{1}{7}\frac{6}{7}\frac{\bar{1}}{7}\frac{\bar{1}}{7}/\frac{1}{7}\frac{1}{7}\frac{1}{7}\frac{1}{7}\frac{\bar{1}}{7}\frac{6}{7}\frac{\bar{1}}{7}/\frac{1}{7}\frac{1}{7}\frac{1}{7}\frac{1}{7}\frac{\bar{1}}{7}\frac{\bar{1}}{7}\frac{6}{7}\right)$$

$PD_1^{\perp} =$

$$\left(\frac{9}{14}\frac{\bar{5}}{14}\frac{1}{7}\frac{1}{7}\frac{\bar{1}}{7}\frac{\bar{1}}{7}\frac{\bar{1}}{7}/\frac{\bar{5}}{14}\frac{9}{14}\frac{1}{7}\frac{1}{7}\frac{\bar{1}}{7}\frac{\bar{1}}{7}\frac{\bar{1}}{7}/\frac{1}{7}\frac{1}{7}\frac{1}{7}\frac{1}{7}\frac{\bar{1}}{7}\frac{\bar{1}}{7}\frac{\bar{1}}{7}/\frac{1}{7}\frac{1}{7}\frac{1}{7}\frac{1}{7}\frac{\bar{1}}{7}\frac{\bar{1}}{7}\frac{\bar{1}}{7}/\frac{\bar{1}}{7}\frac{\bar{1}}{7}\frac{\bar{1}}{7}\frac{\bar{1}}{7}\frac{1}{7}\frac{1}{7}\frac{1}{7}/\frac{\bar{1}}{7}\frac{\bar{1}}{7}\frac{\bar{1}}{7}\frac{\bar{1}}{7}\frac{1}{7}\frac{1}{7}\frac{1}{7}/\frac{\bar{1}}{7}\frac{\bar{1}}{7}\frac{\bar{1}}{7}\frac{\bar{1}}{7}\frac{1}{7}\frac{1}{7}\frac{1}{7}\right)$$

$\mathbf{D^7}$ subspace: $(r, r, s, t, u, v, 2r + s + t - u - v)$

B.7.2. Case 2 $d_2 = d_3$, $R + T = Q + U$, $||\,\mathbf{b}\,||^2 = ||\,\mathbf{c}\,||^2$, $\mathbf{a} \to -\mathbf{a}$, $\mathbf{b} \to -\mathbf{c}$, $\mathbf{c} \to -\mathbf{b}$, $\mathbf{d} \to \mathbf{a} + \mathbf{b} + \mathbf{b} = -\mathbf{d}$

$MD_2 =$ (1000000/0**01**0000/0**10**0000/0001000/0000100/00000**01**/00000**10**)

$PD_2 =$

$$\left(\frac{6}{7}\frac{\bar{1}}{7}\frac{\bar{1}}{7}\frac{\bar{1}}{7}\frac{1}{7}\frac{1}{7}\frac{1}{7}/\frac{\bar{1}}{7}\frac{5}{14}\frac{5}{14}\frac{\bar{1}}{7}\frac{1}{7}\frac{1}{7}\frac{1}{7}/\frac{\bar{1}}{7}\frac{5}{14}\frac{5}{14}\frac{\bar{1}}{7}\frac{1}{7}\frac{1}{7}\frac{1}{7}/\frac{\bar{1}}{7}\frac{\bar{1}}{7}\frac{\bar{1}}{7}\frac{6}{7}\frac{1}{7}\frac{1}{7}\frac{1}{7}/\frac{1}{7}\frac{1}{7}\frac{1}{7}\frac{1}{7}\frac{6}{7}\frac{\bar{1}}{7}\frac{\bar{1}}{7}/\frac{1}{7}\frac{1}{7}\frac{1}{7}\frac{1}{7}\frac{\bar{1}}{7}\frac{6}{7}\frac{\bar{1}}{7}/\frac{1}{7}\frac{1}{7}\frac{1}{7}\frac{1}{7}\frac{\bar{1}}{7}\frac{\bar{1}}{7}\frac{6}{7}\right)$$

$PD_2^{\perp} =$

$$\left(\frac{1}{7}\frac{1}{7}\frac{1}{7}\frac{1}{7}\frac{\bar{1}}{7}\frac{\bar{1}}{7}\frac{\bar{1}}{7}/\frac{1}{7}\frac{9}{14}\frac{\bar{5}}{14}\frac{1}{7}\frac{\bar{1}}{7}\frac{\bar{1}}{7}\frac{\bar{1}}{7}/\frac{1}{7}\frac{\bar{5}}{14}\frac{9}{14}\frac{1}{7}\frac{\bar{1}}{7}\frac{\bar{1}}{7}\frac{\bar{1}}{7}/\frac{1}{7}\frac{1}{7}\frac{1}{7}\frac{1}{7}\frac{\bar{1}}{7}\frac{\bar{1}}{7}\frac{\bar{1}}{7}/\frac{\bar{1}}{7}\frac{\bar{1}}{7}\frac{\bar{1}}{7}\frac{\bar{1}}{7}\frac{1}{7}\frac{1}{7}\frac{1}{7}/\frac{\bar{1}}{7}\frac{\bar{1}}{7}\frac{\bar{1}}{7}\frac{\bar{1}}{7}\frac{1}{7}\frac{1}{7}\frac{1}{7}/\frac{\bar{1}}{7}\frac{\bar{1}}{7}\frac{\bar{1}}{7}\frac{\bar{1}}{7}\frac{1}{7}\frac{1}{7}\frac{1}{7}\right)$$

$\mathbf{D^7}$ subspace: $(r, s, s, t, u, v, r + 2s + t - u - v)$

B.7.3. Case 3 $d_3 = d_4$, $P + Q = S + T$, $||\,\mathbf{c}\,||^2 = ||\,\mathbf{d}\,||^2 = ||\,\mathbf{a}+\mathbf{b}+\mathbf{c}\,||^2$, $\mathbf{a} \to -\mathbf{a}$, $\mathbf{b} \to -\mathbf{b}$, $\mathbf{c} \to \mathbf{a} + \mathbf{b} + \mathbf{c}$, $\mathbf{d} \to -\mathbf{c}$

$MD_3 =$ (1000000/0100000/00**01**000/00**10**000/0000**01**0/0000**10**0/0000001)

$PD_3 =$

$$\left(\frac{6}{7}\frac{\bar{1}}{7}\frac{\bar{1}}{7}\frac{\bar{1}}{7}\frac{1}{7}\frac{1}{7}\frac{1}{7}/\frac{\bar{1}}{7}\frac{6}{7}\frac{\bar{1}}{7}\frac{\bar{1}}{7}\frac{1}{7}\frac{1}{7}\frac{1}{7}/\frac{\bar{1}}{7}\frac{\bar{1}}{7}\frac{5}{14}\frac{5}{14}\frac{1}{7}\frac{1}{7}\frac{1}{7}/\frac{\bar{1}}{7}\frac{\bar{1}}{7}\frac{5}{14}\frac{5}{14}\frac{1}{7}\frac{1}{7}\frac{1}{7}/\frac{1}{7}\frac{1}{7}\frac{1}{7}\frac{1}{7}\frac{6}{7}\frac{\bar{1}}{7}\frac{\bar{1}}{7}/\frac{1}{7}\frac{1}{7}\frac{1}{7}\frac{1}{7}\frac{\bar{1}}{7}\frac{6}{7}\frac{\bar{1}}{7}/\frac{1}{7}\frac{1}{7}\frac{1}{7}\frac{1}{7}\frac{\bar{1}}{7}\frac{\bar{1}}{7}\frac{6}{7}\right)$$

$PD_3^{\perp} =$

$$\left(\frac{1}{7}\frac{1}{7}\frac{1}{7}\frac{1}{7}\frac{\overline{1}}{7}\frac{\overline{1}}{7}\frac{\overline{1}}{7}/\frac{1}{7}\frac{1}{7}\frac{1}{7}\frac{1}{7}\frac{\overline{1}}{7}\frac{\overline{1}}{7}\frac{\overline{1}}{7}/\frac{1}{7}\frac{1}{7}\frac{9}{14}\frac{\overline{5}}{14}\frac{\overline{1}}{7}\frac{\overline{1}}{7}\frac{\overline{1}}{7}/\frac{1}{7}\frac{1}{7}\frac{\overline{5}}{14}\frac{9}{14}\frac{\overline{1}}{7}\frac{\overline{1}}{7}\frac{\overline{1}}{7}/\frac{\overline{1}}{7}\frac{\overline{1}}{7}\frac{\overline{1}}{7}\frac{\overline{1}}{7}\frac{1}{7}\frac{1}{7}\frac{1}{7}/\frac{\overline{1}}{7}\frac{\overline{1}}{7}\frac{\overline{1}}{7}\frac{\overline{1}}{7}\frac{1}{7}\frac{1}{7}\frac{1}{7}/\frac{\overline{1}}{7}\frac{\overline{1}}{7}\frac{\overline{1}}{7}\frac{\overline{1}}{7}\frac{1}{7}\frac{1}{7}\frac{1}{7}\right)$$

$\mathbf{D^7}$ subspace: $(r, s, t, t, u, v, r + s + 2t - u - v)$

B.7.4. Other equality cases Consider the other possible edge length equality cases. The other potential boundary polytopes to consider are:

$$d_1 = d_3 \tag{B.16}$$

$$d_1 = d_4 \tag{B.17}$$

$$d_1 = d_5 \tag{B.18}$$

$$d_1 = d_6 \tag{B.19}$$

$$d_1 = d_7 \tag{B.20}$$

$$d_2 = d_4 \tag{B.21}$$

$$d_2 = d_5 \tag{B.22}$$

$$d_2 = d_6 \tag{B.23}$$

$$d_2 = d_7 \tag{B.24}$$

$$d_3 = d_5 \tag{B.25}$$

$$d_3 = d_6 \tag{B.26}$$

$$d_3 = d_7 \tag{B.27}$$

$$d_4 = d_5 \tag{B.28}$$

$$d_4 = d_6 \tag{B.29}$$

$$d_4 = d_7 \tag{B.30}$$

$$d_5 = d_6 \tag{B.31}$$

$$d_5 = d_7 \tag{B.32}$$

$$d_6 = d_7 \tag{B.33}$$

The boundary polytopes B.16, B.17, and B.21 are of dimensions 4, 3 and 4, respectively, because they imply additional equalities from the ordering in B.3. Many of the others arise in mapping Niggli characters into Delone conditions.

B.8. Cases 4, 5, 6, 7, 8, 9: Right angle cases

There are six cases, cases 4, 5, 6, 7, 8, 9 given by expressions B.4 through B.9 with inequality replaced by equality, in which edges of the Bravais tetrahedron meet at right angles. Cases 4, 5, 6, and 8 are 5-dimensional and cases 7 and 9 are of lower dimension. Consider for example

$$d_5 = d_2 + d_3$$

$$g_2 + g_3 + g_4 = g_2 + g_3$$

$$g_4 = 2\mathbf{b} \cdot \mathbf{c} = 0$$

The full set of resulting equations in the same ordering as expressions B.4 through B.9 are

$$2\mathbf{b} \cdot \mathbf{c} = g_4 = 0; P = 0 \tag{B.34}$$

$$2\mathbf{a}\cdot\mathbf{d} = 2\mathbf{a}\cdot(-\mathbf{a}-\mathbf{b}-\mathbf{c}) = -2g_1 - g_6 - g_5 = 0; S = 0 \tag{B.35}$$

$$2\mathbf{a}\cdot\mathbf{c} = g_5 = 0; Q = 0 \tag{B.36}$$

$$2\mathbf{b}\cdot\mathbf{d} = 2\mathbf{b}\cdot(-\mathbf{a}-\mathbf{b}-\mathbf{c}) = -g_6 - 2g_2 - g_4 = 0; T = 0; \tag{B.37}$$

$$2\mathbf{a}\cdot\mathbf{b} = g_6 = 0; R = 0 \tag{B.38}$$

$$2\mathbf{c}\cdot\mathbf{d} = 2\mathbf{c}\cdot(-\mathbf{a}-\mathbf{b}-\mathbf{c}) = -g_5 - g_4 - 2g_3 = 0; U = 0; \tag{B.39}$$

The right angle cases are Delone reduced. However a slight perturbation will introduce an acute angle, necessitating a transformation to return to $\mathbf{D^7}$. In order to understand the necessary transformation, consider a Delone-reduced cell with tetrahedron

$$\mathbf{a}_{orig}, \mathbf{b}_{orig}, \mathbf{c}_{orig}, -\mathbf{a}_{orig} - \mathbf{b}_{orig} - \mathbf{c}_{orig}$$

for which $2\mathbf{b}_{orig}\cdot\mathbf{c}_{orig} = 0$. Impose a slight perturbation on $\mathbf{b}_{orig}$ to form $\mathbf{b}_{ptrb}$ such that $\mathbf{b}_{ptrb}\cdot\mathbf{c}_{orig} = \epsilon > 0$, and such that all other inner products remain non-positive. We convert from the $+--$ presentation to $+++$ by changing +the sign of $\mathbf{a}$. If we define $\mathbf{a}_{ptrb} = -\mathbf{a}_{orig}$ with a small additional perturbation to guarantee that

$$2\mathbf{a}_{ptrb}\cdot\mathbf{c}_{orig} > 0$$

$$2\mathbf{a}_{ptrb}\cdot\mathbf{b}_{ptrb} > 0$$

then we are starting from a $+++$ case with a small g_4 and can apply the transformation in expression A.9 to return to the $---$ case using the tetrahedron

$$\mathbf{b}_{ptrb} - \mathbf{a}_{ptrb}, -\mathbf{b}_{ptrb}, \mathbf{c}_{orig}, \mathbf{a}_{ptrb} - \mathbf{c}_{orig}$$

which says, up to reordering, if the $+++$ case we generated is Niggli-reduced, the boundary transform takes the tetrahedron to

$$\mathbf{b}+\mathbf{a}, -\mathbf{b}, \mathbf{c}, -\mathbf{a}-\mathbf{c}$$

There is only one mapping back into $\mathbf{D^7}$ for each right angle case, but in each of these cases several permuted versions of the boundary transform may be needed to ensure that the results are ordered $d_1 \le d_2 \le d_3 \le d_4$. While there are, in general, 24 permutations of 4 objects, the ordering constraint reduces the number of acceptable permutations to eight, six, or zero cases that represent 5-dimensional boundaries. The remaining permutations imply additional constraints that lower the dimensionality of the resulting boundary polytope.

The general pattern is that the new Bravais tetrahedron resulting from a right-angle boundary mapping will change the sign of one of the two edges involved in the right angle and leave the other edge that is involved unchanged.

B.8.1. Case 4: $d_5 = d_2 + d_3$ (see equation B.4). This is equivalent to $g_4 = 0$. The Bravais tetrahedron edges to be ordered are

$$\mathbf{a} + \mathbf{b}, -\mathbf{b}, \mathbf{c}, -\mathbf{a} - \mathbf{c}$$

In this case, the only acceptable permutations are ones that preserve the relative ordering of $||\,\mathbf{a}\,||^2 \le ||\,\mathbf{b}\,||^2 \le ||\,\mathbf{c}\,||^2 \le ||\,\mathbf{d}\,||^2$ with obtuse angles. If $||\,\mathbf{b}\,||^2$ is presented first, all six permutations of the remaining three edges are feasible. It is not possible to present $||\,\mathbf{c}\,||^2$ first, because that would leave no room to present $||\,\mathbf{b}\,||^2$, except in the lower-dimensional polytope resulting from the intersection of Case 4 with Case 2.

If $||\,\mathbf{a}+\mathbf{b}\,||^2$ is presented first, then we must have $||\,\mathbf{a}+\mathbf{b}\,||^2 \le ||-\mathbf{b}\,||^2$ which is equivalent to

$$||\,\mathbf{a}\,||^2 + 2\mathbf{a}\cdot\mathbf{b} \le 0 \tag{B.40}$$

From the ordering constraint $||\,\mathbf{c}\,||^2 \le ||\,\mathbf{d}\,||^2$ and equation B.40 it follows that

$$0 \le ||\,\mathbf{a}\,||^2 + ||\,\mathbf{b}\,||^2 + 2\mathbf{b}\cdot\mathbf{c} + 2\mathbf{a}\cdot\mathbf{c} + 2\mathbf{a}\cdot\mathbf{b} \le ||\,\mathbf{b}\,||^2 + 2\mathbf{b}\cdot\mathbf{c} + 2\mathbf{a}\cdot\mathbf{c} = ||-\mathbf{a}-\mathbf{c}|| - ||\,\mathbf{c}\,||^2 + 2\mathbf{b}\cdot\mathbf{c}$$

but from the obtuseness of the angles in a Bravais tetrahedron, we have

$$|| - \mathbf{a} - \mathbf{c}|| - ||\,\mathbf{c}\,||^2 \geq 0$$

i.e.

$$|| - \mathbf{a} - \mathbf{c}|| \geq ||\,\mathbf{c}\,||^2$$

which allows only one ordering in this case. Similarly there is only one ordering when $||\,\mathbf{a} + \mathbf{c}\,||^2$ is presented first. Thus there is a total of eight 5-dimensional cases:

$$MD_{4.1} = (0100000/0010000/0000010/0000001/1000000/0001000/0220\overline{1}00)$$

$$MD_{4.2} = (0100000/0010000/0000001/0000010/0001000/1000000/0220\overline{1}00)$$

$$MD_{4.3} = (0100000/0000010/0010000/0000001/1000000/0220\overline{1}00/0001000)$$

$$MD_{4.4} = (0100000/0000010/0000001/0010000/0220\overline{1}00/1000000/0001000)$$

$$MD_{4.5} = (0100000/0000001/0010000/0000010/0001000/0220\overline{1}00/1000000)$$

$$MD_{4.6} = (0100000/0000001/0000010/0010000/0220\overline{1}00/0001000/1000000)$$

$$MD_{4.7} = (0000010/0100000/0010000/0000001/0220\overline{1}00/1000000/0001000)$$

$$MD_{4.8} = (0000001/0100000/0010000/0000010/0220\overline{1}00/0001000/1000000)$$

$PD_4 =$

$$\left(\frac{3}{4}00\frac{\bar{1}}{4}0\frac{1}{4}\frac{1}{4}/0\frac{2}{3}\frac{\bar{1}}{3}0\frac{1}{3}00/0\frac{\bar{1}}{3}\frac{2}{3}0\frac{1}{3}00/\frac{\bar{1}}{4}00\frac{3}{4}0\frac{1}{4}\frac{1}{4}/0\frac{1}{3}\frac{1}{3}0\frac{2}{3}00/\frac{1}{4}00\frac{1}{4}0\frac{3}{4}\frac{\bar{1}}{4}/\frac{1}{4}00\frac{1}{4}0\frac{\bar{1}}{4}\frac{3}{4}\right)$$

$PD_4^{\perp} =$

$$\left(\frac{1}{4}00\frac{1}{4}0\frac{\bar{1}}{4}\frac{\bar{1}}{4}/0\frac{1}{3}\frac{1}{3}0\frac{\bar{1}}{3}00/0\frac{1}{3}\frac{1}{3}0\frac{\bar{1}}{3}00/\frac{1}{4}00\frac{1}{4}0\frac{\bar{1}}{4}\frac{\bar{1}}{4}/0\frac{\bar{1}}{3}\frac{\bar{1}}{3}0\frac{1}{3}00/\frac{\bar{1}}{4}00\frac{\bar{1}}{4}0\frac{1}{4}\frac{1}{4}/\frac{\bar{1}}{4}00\frac{\bar{1}}{4}0\frac{1}{4}\frac{1}{4}\right)$$

This implies $\mathbf{D^7}$ cells of the form $[r, s, t, u, s+t, v, r+u-v]$, $0 \leq r \leq s \leq t \leq u \leq r+v$ $v \leq s+u$. As $\mathbf{G^6}$ cells, these cells are of the form $[r, s, t, 0, v-t-r, u-v-s]$.

B.8.2. Case 4 Internal Boundaries The eight permutations that constitute the case 4 five-dimensional case in terms of $\mathbf{D^7}$ components are

d_2, d_3, d_6, d_7

d_2, d_3, d_7, d_6

d_2, d_6, d_3, d_7

d_2, d_6, d_7, d_3

d_2, d_7, d_3, d_6

d_2, d_7, d_6, d_3

d_6, d_2, d_3, d_7

d_7, d_2, d_3, d_6

so the internal boundaries are:

$\{4.1, 4.2\} : d_6 = d_7$

$\{4.1, 4.3\} : d_3 = d_6$

$\{4.3, 4.4\} : d_3 = d_7$

$\{4.2, 4.5\} : d_3 = d_7$

$\{4.4, 4.6\} : d_6 = d_7$

$\{4.3, 4.7\} : d_2 = d_6$

$\{4.5, 4.8\} : d_2 = d_7$

leaving the conditions $d_6 = d_7$, $d_3 = d_6$, $d_3 = d_7$, $d_2 = d_6$, and $d_2 = d_7$, all subject to the case 4 conditions $d_5 = d_2 + d_3$, $g_4 = 0$ and the general Bravais tetrahedron condition $d_1 + d_2 + d_3 + d_4 - d_5 - d_6 - d_7$ to analyze.

The projectors onto the 4-dimensional internal boundaries are:

$PD_{4\_67} =$

$$\left(\frac{3}{4}00\frac{\overline{1}}{4}0\frac{1}{4}\frac{1}{4}/0\frac{2}{3}\frac{\overline{1}}{3}0\frac{1}{3}00/0\frac{\overline{1}}{3}\frac{2}{3}0\frac{1}{3}00/\frac{\overline{1}}{4}00\frac{3}{4}0\frac{1}{4}\frac{1}{4}/0\frac{1}{3}\frac{1}{3}0\frac{2}{3}00/\frac{1}{4}00\frac{1}{4}0\frac{1}{4}\frac{1}{4}/\frac{1}{4}00\frac{1}{4}0\frac{1}{4}\frac{1}{4}\right)$$

$PD_{4\_36} =$

$$(\frac{12}{17}\frac{\overline{1}}{17}\frac{2}{17}\frac{\overline{5}}{17}\frac{1}{17}\frac{2}{17}\frac{5}{17}/\frac{\overline{1}}{17}\frac{10}{17}\frac{\overline{3}}{17}\frac{\overline{1}}{17}\frac{7}{17}\frac{\overline{3}}{17}\frac{1}{17}/\frac{2}{17}\frac{\overline{3}}{17}\frac{6}{17}\frac{2}{17}\frac{3}{17}\frac{6}{17}\frac{\overline{2}}{17}/\frac{\overline{5}}{17}\frac{\overline{1}}{17}\frac{2}{17}\frac{12}{17}\frac{1}{17}\frac{2}{17}\frac{5}{17}$$
$$\frac{1}{17}\frac{7}{17}\frac{3}{17}\frac{1}{17}\frac{10}{17}\frac{3}{17}\frac{\overline{1}}{17}/\frac{2}{17}\frac{\overline{3}}{17}\frac{6}{17}\frac{2}{17},\frac{3}{17}\frac{6}{17}\frac{\overline{2}}{17}/\frac{5}{17}\frac{1}{17}\frac{\overline{2}}{17}\frac{5}{17}\frac{\overline{1}}{17}\frac{\overline{2}}{17}\frac{12}{17})$$

$PD_{4\_37} =$

$$(\frac{12}{17}\frac{\overline{1}}{17}\frac{2}{17}\frac{\overline{5}}{17}\frac{1}{17}\frac{5}{17}\frac{2}{17}/\frac{\overline{1}}{17}\frac{10}{17}\frac{\overline{3}}{17}\frac{\overline{1}}{17}\frac{7}{17}\frac{1}{17}\frac{\overline{3}}{17}/\frac{2}{17}\frac{\overline{3}}{17}\frac{6}{17}\frac{2}{17}\frac{3}{17}\frac{\overline{2}}{17}\frac{6}{17}/\frac{\overline{5}}{17}\frac{\overline{1}}{17}\frac{2}{17}\frac{12}{17}\frac{1}{17}\frac{5}{17}\frac{2}{17}$$
$$\frac{1}{17}\frac{7}{17}\frac{3}{17}\frac{1}{17}\frac{10}{17}\frac{\overline{1}}{17}\frac{3}{17}/\frac{5}{17}\frac{1}{17}\frac{\overline{2}}{17}\frac{5}{17}\frac{\overline{1}}{17}\frac{12}{17}\frac{\overline{2}}{17}/\frac{2}{17}\frac{\overline{3}}{17}\frac{6}{17}\frac{2}{17}\frac{3}{17}\frac{\overline{2}}{17}\frac{6}{17})$$

$PD_{4\_26} =$

$$(\frac{12}{17}\frac{2}{17}\frac{\overline{1}}{17}\frac{\overline{5}}{17}\frac{1}{17}\frac{2}{17}\frac{5}{17}/\frac{2}{17}\frac{6}{17}\frac{\overline{3}}{17}\frac{2}{17}\frac{3}{17}\frac{6}{17}\frac{\overline{2}}{17}/\frac{\overline{1}}{17}\frac{\overline{3}}{17}\frac{10}{17}\frac{\overline{1}}{17}\frac{7}{17}\frac{\overline{3}}{17}\frac{1}{17}/\frac{\overline{5}}{17}\frac{2}{17}\frac{\overline{1}}{17}\frac{12}{17}\frac{1}{17}\frac{2}{17}\frac{5}{17}$$
$$\frac{1}{17}\frac{3}{17}\frac{7}{17}\frac{1}{17}\frac{10}{17}\frac{3}{17}\frac{\overline{1}}{17}/\frac{2}{17}\frac{6}{17}\frac{\overline{3}}{17}\frac{2}{17}\frac{3}{17}\frac{6}{17}\frac{\overline{2}}{17}/\frac{5}{17}\frac{\overline{2}}{17}\frac{1}{17}\frac{5}{17}\frac{\overline{1}}{17}\frac{\overline{2}}{17}\frac{12}{17})$$

$PD_{4\_27} =$

$$(\frac{12}{17}\frac{2}{17}\frac{\overline{1}}{17}\frac{\overline{5}}{17}\frac{1}{17}\frac{5}{17}\frac{2}{17}/\frac{2}{17}\frac{6}{17}\frac{\overline{3}}{17}\frac{2}{17}\frac{3}{17}\frac{\overline{2}}{17}\frac{6}{17}/\frac{\overline{1}}{17}\frac{\overline{3}}{17}\frac{10}{17}\frac{\overline{1}}{17}\frac{7}{17}\frac{1}{17}\frac{\overline{3}}{17}/\frac{\overline{5}}{17}\frac{2}{17}\frac{\overline{1}}{17}\frac{12}{17}\frac{1}{17}\frac{5}{17}\frac{2}{17}$$
$$\frac{1}{17}\frac{3}{17}\frac{7}{17}\frac{1}{17}\frac{10}{17}\frac{\overline{1}}{17}\frac{3}{17}/\frac{5}{17}\frac{\overline{2}}{17}\frac{1}{17}\frac{5}{17}\frac{\overline{1}}{17}\frac{12}{17}\frac{\overline{2}}{17}/\frac{2}{17}\frac{6}{17}\frac{\overline{3}}{17}\frac{2}{17}\frac{3}{17}\frac{\overline{2}}{17}\frac{6}{17})$$

B.8.3. Case 5: $d_5 = d_1 + d_4$ (see equation B.5). The Bravais tetrahedron edges to be ordered are

$$-\mathbf{a}, \mathbf{a}+\mathbf{b}, \mathbf{a}+\mathbf{c}, \mathbf{d} = -\mathbf{a}-\mathbf{b}-\mathbf{c}$$

As in case 4, above, the original Bravais tetrahedron edge ordering must be respected in selecting permutations, which limits us to permutations that begin with $||-\mathbf{a}||^2$, $||\mathbf{a}+\mathbf{b}||^2$ or $||\mathbf{a}+\mathbf{c}||^2$. If we were to begin with $||\mathbf{d}||^2$ there would be no room for $||-\mathbf{a}||^2$ except in the lower-dimensional case of the intersection of case 5 with cases 1, 2 and 3.

Consider the permutations that begin with $||-\mathbf{a}||^2$. There are six possible permtations of $||\mathbf{d}||^2$, $||\mathbf{a}+\mathbf{b}||^2$, and $||\mathbf{a}+\mathbf{c}||^2$ to consider. If $||-\mathbf{a}||^2$ does not come first, then it must come second and only either $||\mathbf{a}+\mathbf{b}||^2$ or $||\mathbf{a}+\mathbf{c}||^2$ may come before it or we are forced into lower-dimensional cases.

$$MD_{5.1} = (1000000/0001000/0000010/0000001/0100000/0010000/2002\bar{1}00)$$

$$MD_{5.2} = (1000000/0001000/0000001/0000010/0010000/0100000/2002\bar{1}00)$$

$$MD_{5.3} = (1000000/0000010/0001000/0000001/0100000/2002\bar{1}00/0010000)$$

$$MD_{5.4} = (1000000/0000010/0000001/0001000/2002\bar{1}00/0100000/0010000)$$

$$MD_{5.5} = (1000000/0000001/0001000/0000010/0010000/2002\bar{1}00/0100000)$$

$$MD_{5.6} = (1000000/0000001/0000010/0001000/2002\bar{1}00/0010000/0100000)$$

$$MD_{5.7} = (0000010/1000000/0001000/0000001/2002\bar{1}00/0100000/0010000)$$

$$MD_{5.8} = (0000001/1000000/0001000/0000010/2002\bar{1}00/0010000/0100000)$$

$$PD_5 =$$

$$\left(\frac{2}{3}00\frac{\bar{1}}{3}\frac{1}{3}00/0\frac{3}{4}\frac{\bar{1}}{4}00\frac{1}{4}\frac{1}{4}/0\frac{\bar{1}}{4}\frac{3}{4}00\frac{1}{4}\frac{1}{4}/\frac{\bar{1}}{3}00\frac{2}{3}\frac{1}{3}00/\frac{1}{3}00\frac{1}{3}\frac{2}{3}00/0\frac{1}{4}\frac{1}{4}00\frac{3}{4}\frac{\bar{1}}{4}/0\frac{1}{4}\frac{1}{4}00\frac{\bar{1}}{4}\frac{3}{4}\right)$$

$$PD_5^{\perp} =$$

$$\left(\frac{1}{3}00\frac{1}{3}\frac{\bar{1}}{3}00/0\frac{1}{4}\frac{1}{4}00\frac{\bar{1}}{4}\frac{\bar{1}}{4}/0\frac{1}{4}\frac{1}{4}00\frac{\bar{1}}{4}\frac{\bar{1}}{4}/\frac{1}{3}00\frac{1}{3}\frac{\bar{1}}{3}00/\frac{\bar{1}}{3}00\frac{\bar{1}}{3}\frac{1}{3}00/0\frac{\bar{1}}{4}\frac{\bar{1}}{4}00\frac{1}{4}\frac{1}{4}/0\frac{\bar{1}}{4}\frac{\bar{1}}{4}00\frac{1}{4}\frac{1}{4}\right)$$

This implies $\mathbf{D^7}$ cells of the form $[r, s, t, u, r+u, v, s+t-v]$, $0 \le r \le s \le t \le u$, $u+r \le t+s$, $t \le r+v$, $v \le r+t$, which are $\mathbf{G^6}$ cells of the form $[r, s, t, u-t-s+r, v-t-r, t-r-v]$.

B.8.4. Case 6: $d_6 = d_1 + d_3$, (see equation B.6). The Bravais tetrahedron edges to be ordered are

$$\mathbf{a}, -\mathbf{a}-\mathbf{b}, -\mathbf{c}, \mathbf{b}+\mathbf{c}$$

As with case 4, there are 8 permutations that result in 5-dimensional boundary polytopes.

$$MD_{6.1} = (1000000/0010000/0000100/0000001/0100000/0001000/20200\bar{1}0)$$

$$MD_{6.2} = (1000000/0010000/0000001/0000100/0001000/0100000/20200\bar{1}0)$$

$$MD_{6.3} = (1000000/0000100/0010000/0000001/0100000/20200\bar{1}0/0001000)$$

$$MD_{6.4} = (1000000/0000100/0000001/0010000/20200\bar{1}0/0100000/0001000)$$

$$MD_{6.5} = (1000000/0000001/0010000/0000100/0001000/20200\bar{1}0/0100000)$$

$$MD_{6.6} = (1000000/0000001/0000100/0010000/20200\bar{1}0/0001000/0100000)$$

$$MD_{6.7} = (0000100/1000000/0010000/0000001/20200\bar{1}0/0100000/0001000)$$

$$MD_{6.8} = (0000001/1000000/0010000/0000100/20200\bar{1}0/0001000/0100000)$$

$PD_6 =$

$$\left(\frac{2}{3}0\frac{\bar{1}}{3}00\frac{1}{3}0/0\frac{3}{4}0\frac{\bar{1}}{4}\frac{1}{4}0\frac{1}{4}/\frac{\bar{1}}{3}0\frac{2}{3}00\frac{1}{3}0/0\frac{\bar{1}}{4}0\frac{3}{4}\frac{1}{4}0\frac{1}{4}/0\frac{1}{4}0\frac{1}{4}\frac{3}{4}0\frac{\bar{1}}{4}/\frac{1}{3}0\frac{1}{3}00\frac{2}{3}0/0\frac{1}{4}0\frac{1}{4}\frac{\bar{1}}{4}0\frac{3}{4}\right)$$

$PD_6^{\perp} =$

$$\left(\frac{1}{3}0\frac{1}{3}00\frac{\bar{1}}{3}0/0\frac{1}{4}0\frac{1}{4}\frac{\bar{1}}{4}0\frac{\bar{1}}{4}/\frac{1}{3}0\frac{1}{3}00\frac{\bar{1}}{3}0/0\frac{1}{4}0\frac{1}{4}\frac{\bar{1}}{4}0\frac{\bar{1}}{4}/0\frac{\bar{1}}{4}0\frac{\bar{1}}{4}\frac{1}{4}0\frac{1}{4}/\frac{\bar{1}}{3}0\frac{\bar{1}}{3}00\frac{1}{3}0/0\frac{\bar{1}}{4}0\frac{\bar{1}}{4}\frac{1}{4}0\frac{1}{4}\right)$$

This implies $\mathbf{D^7}$ cells of the form $[r, s, t, u, v, r+t, s+u-v]$, $0 \leq r \leq s \leq t \leq u \leq r+v$, $v \leq s+u$, which are $\mathbf{G^6}$ cells of the form $[r, s, t, v-t-s, 0, u-v-r]$.

B.8.5. Case 7, a subboundary of Case 6: $d_6 = d_2 + d_4$ (see equation B.7). The Bravais tetrahedron edges to be ordered are

$$\mathbf{a}+\mathbf{b}, -\mathbf{b}, \mathbf{b}+\mathbf{c}, \mathbf{d} = -\mathbf{a}-\mathbf{b}-\mathbf{c}$$

This is not a 5-dimensional boundary. If we call the original case 7 boundary Bravais tetrahedron

$$\mathbf{a_7}, \mathbf{b_7}, \mathbf{c_7}, \mathbf{d_7} = -\mathbf{a_7} - \mathbf{b_7} - \mathbf{c_7}$$

with the ordering

$$|| \mathbf{a_7} ||^2 \leq || \mathbf{b_7} ||^2 \leq || \mathbf{c_7} ||^2 \leq || \mathbf{d_7} ||^2$$

which, by subtracting $|| \mathbf{c_7} ||^2$ from the third and fourth terms implies

$$0 \leq || \mathbf{a_7} ||^2 + || \mathbf{b_7} ||^2 + 2\mathbf{b_7} \cdot \mathbf{c_7} + 2\mathbf{a_7} \cdot \mathbf{c_7} + 2\mathbf{a_7} \cdot \mathbf{b_7}$$

$$= (|| \mathbf{a_7} ||^2 - || \mathbf{b_7} ||^2) + (|| \mathbf{b_7} ||^2 - || \mathbf{b_7} ||^2) + (2\mathbf{a_7} \cdot \mathbf{c_7}) + (2|| \mathbf{b_7} ||^2 + 2\mathbf{a_7} \cdot \mathbf{b_7} + 2\mathbf{b_7} \cdot \mathbf{c_7})$$

in which each of the parenthesized terms is less than or equal to zero, which means each of them is, indeed, equal to zero, so that

$$|| \mathbf{a_7} ||^2 = || \mathbf{b_7} ||^2$$

and

$$2\mathbf{a_7} \cdot \mathbf{c_7} = 0$$

then this all, combined with $d_6 = d_1 + d_3$, gives three constraints, thereby lowering the dimension of this boundary to three.

The three-dimensional projector, with “C” in place of “12” and “D” in place of “13” is:

$$PD_7 = \frac{(77\overline{33}242/77\overline{33}242/\overline{33}77242/\overline{33}77242/2222C4\overline{8}/4444484/2222\overline{8}4C)}{20}$$

$$PD_7^{\perp} = \frac{(D\overline{7}33\overline{242}/\overline{7}D33\overline{242}/33D\overline{7242}/33D\overline{7242}/\overline{2222}8\overline{4}8/\overline{44444}C\overline{4}/\overline{2222}8\overline{4}8)}{20}$$

This implies $\mathbf{D^7}$ cells of the form $[r, r, s, s, t, r+s, r+s-t]$, $0 \leq r \leq s$ $0 \leq t \leq r+s$, $s \leq r+t$, which are $\mathbf{G^6}$ cells of the form $[r, r, s, t-s-r, 0, -t+s-r]$. Thus all case 7 cells are also case 6 cells.

B.8.6. Case 8: $d_7 = d_1 + d_2$ (see equation B.8). This is equivalent to $g_6 = 0$. The Bravais tetrahedron edges to be ordered are

$$\mathbf{a}, -\mathbf{b}, \mathbf{b}+\mathbf{c}, -\mathbf{a}-\mathbf{c}$$

As in case 4, above, the original Bravais tetrahedron edge ordering must be respected in selecting permutations, which requires that $||a||^2 \leq ||b||^2$. In addition, neither $||\,\mathbf{a}\,||^2$ nor $||\,\mathbf{b}\,||^2$ can be larger than both $||\,\mathbf{b}+\mathbf{c}\,||^2$ and $||-\mathbf{a}-\mathbf{c}\,||^2$ or we will force $||\,\mathbf{d}\,||^2$ to be equal to $||\,\mathbf{a}\,||^2$ or $||\,\mathbf{b}\,||^2$, respectively, thereby lowering the dimension of the boundary. This leaves the following six feasible 5-dimensional polytopes.

$$MD_{8.1} = (1000000/0100000/0000100/0000010/0010000/0001000/220000\overline{1})$$

$$MD_{8.2} = (1000000/0100000/0000010/0000100/0001000/0010000/220000\overline{1})$$

$$MD_{8.3} = (1000000/0000100/0100000/0000010/0010000/220000\overline{1}/0001000)$$

$$MD_{8.4} = (1000000/0000010/0100000/0000100/0001000/220000\overline{1}/0010000)$$

$$MD_{8.5} = (0000010/1000000/0100000/0000100/220000\overline{1}/0001000/0010000)$$

$$MD_{8.6} = (0000100/1000000/0100000/0000010/220000\overline{1}/0010000/0001000)$$

$$PD_8 =$$

$$\left(\frac{2}{3}\frac{\bar{1}}{3}0000\frac{1}{3}/\frac{\bar{1}}{3}\frac{2}{3}0000\frac{1}{3}/00\frac{3}{4}\frac{\bar{1}}{4}\frac{1}{4}\frac{1}{4}0/00\frac{\bar{1}}{4}\frac{3}{4}\frac{1}{4}\frac{1}{4}0/00\frac{1}{4}\frac{1}{4}\frac{3}{4}\frac{\bar{1}}{4}0/00\frac{1}{4}\frac{1}{4}\frac{\bar{1}}{4}\frac{3}{4}0/\frac{1}{3}\frac{1}{3}0000\frac{2}{3}\right)$$

$PD_8^{\perp} =$

$$\left(\frac{1}{3}\frac{1}{3}0000\frac{\bar{1}}{3}/\frac{1}{3}\frac{1}{3}0000\frac{\bar{1}}{3}/00\frac{1}{4}\frac{1}{4}\frac{\bar{1}}{4}\frac{\bar{1}}{4}0/00\frac{1}{4}\frac{1}{4}\frac{\bar{1}}{4}\frac{\bar{1}}{4}0/00\frac{\bar{1}}{4}\frac{\bar{1}}{4}\frac{1}{4}\frac{1}{4}0/00\frac{\bar{1}}{4}\frac{\bar{1}}{4}\frac{1}{4}\frac{1}{4}0/\frac{\bar{1}}{3}\frac{\bar{1}}{3}0000\frac{1}{3}\right)$$

This implies $\mathbf{D^7}$ cells of the form $[r, s, t, u, v, t+u-v, r+s]$, $0 \leq r \leq s \leq t \leq u$, $t \leq u - v$, $v \leq s + u$, which are $\mathbf{G^6}$ cells of the form $[r, s, t, v-t-s, u-v-r, 0]$.

B.8.7. Case 9, as subboundary of Case 8: $d_7 = d_3 + d_4$ (see equation B.9). The Bravais tetrahedron edges to be ordered are

$$-\mathbf{c}, \mathbf{a}+\mathbf{c}, \mathbf{b}+\mathbf{c}, \mathbf{d} = -\mathbf{a}-\mathbf{b}-\mathbf{c}$$

This is not a 5-dimensional boundary. If we call case 9 boundary Bravais tetrahedron

$$\mathbf{a_9}, \mathbf{b_9}, \mathbf{c_9}, \mathbf{d_9} = -\mathbf{a_9} - \mathbf{b_9} - \mathbf{c_9}$$

with the ordering

$$||\,\mathbf{a_9}\,||^2 \leq ||\,\mathbf{b_9}\,||^2 \leq ||\,\mathbf{c_9}\,||^2 \leq ||\,\mathbf{d_9}\,||^2$$

which, by subtracting $||\,\mathbf{c_9}\,||^2$ from the third and fourth terms implies

$$0 \leq ||\,\mathbf{a_9}\,||^2 + ||\,\mathbf{b_9}\,||^2 + 2\mathbf{b_9}\cdot\mathbf{c_9} + 2\mathbf{a_9}\cdot\mathbf{c_9} + 2\mathbf{a_9}\cdot\mathbf{b_9}$$

$$= (||\,\mathbf{a_9}\,||^2 - ||\,\mathbf{c_9}\,||^2) + (||\,\mathbf{b_9}\,||^2 - ||\,\mathbf{c_9}\,||^2) + (2\mathbf{a_9}\cdot\mathbf{b_9}) + (2||\,\mathbf{c_9}\,||^2 + 2\mathbf{a_9}\cdot\mathbf{c_9} + 2\mathbf{b_9}\cdot\mathbf{c_9}) \leq 0$$

in which each of the parenthesized terms in less than or equal to zero, which means each of them is, indeed, equal to zero, so that

$$||\,\mathbf{a_9}\,||^2 = ||\,\mathbf{b_9}\,||^2 = ||\,\mathbf{c_9}\,||^2$$

and

$$2\mathbf{a_9}\cdot\mathbf{b_9} = 0$$

then this all, combined with $d_7 = d_3 + d_4$, gives four constraints, thereby lowering the dimension of this boundary to two.

Monte Carlo experiments have not produced any examples of this boundary thus far. If these cases do exist, they should be permutations of

$$MD_{9.1} = (0010000/0000010/0000100/0001000/\overline{2}\overline{2}00221/0100000/1000000)$$

The 2-dimensional projector for case 9 is

$$PD_9 = \frac{(1111112/1111112/1111112/1111112/11116\overline{4}2/1111\overline{4}62/2222224)}{10}$$

$$PD_9^{\perp} = \frac{(9\overline{111112}/\overline{1}9\overline{11112}/\overline{11}9\overline{1112}/\overline{111}9\overline{112}/\overline{1111}44\overline{2}/\overline{1111}44\overline{2}/\overline{222222}6)}{10}$$

This implies $\mathbf{D^7}$ cells of the form $[r, r, r, r, s, 2r - s, 2r]$, $0 \le r$, $0 \le s \le 2r$, which are $\mathbf{G^6}$ cells of the form $[r, r, r, s - 2r, -s, 0]$. Thus all case 9 cells are also case 8 cells.
